# The Need to Prioritize Model-Updating Processes in Clinical Artificial Intelligence (AI) Models: Protocol for a Scoping Review

**DOI:** 10.2196/37685

**Published:** 2023-02-16

**Authors:** Ahmed Umar Otokiti, Makuochukwu Maryann Ozoude, Karmen S Williams, Rasheedat A Sadiq-onilenla, Soji Akin Ojo, Leyla B Wasarme, Samantha Walsh, Maxwell Edomwande

**Affiliations:** 1 Digital Health Solutions, LLC White Plains, NY United States; 2 Zaporozhye State Medical University Zaporizhzhia Ukraine; 3 City University of New York New York, NY United States; 4 Department of Quality Management Elevance Health (Amerigroup Solutions) Iselin, NJ United States; 5 Pharmaceutical Product Development (PPD) Thermo Fisher Scientific Wilmington, NC United States; 6 Geisinger Health Systems Danville, PA United States; 7 Levy Library Icahn School of Medicine at Mount Sinai New York, NY United States; 8 Nuance Communications Inc Burlington, MA United States

**Keywords:** model updating, model calibration, artificial intelligence, machine learning, direct clinical care

## Abstract

**Background:**

With an increase in the number of artificial intelligence (AI) and machine learning (ML) algorithms available for clinical settings, appropriate model updating and implementation of updates are imperative to ensure applicability, reproducibility, and patient safety.

**Objective:**

The objective of this scoping review was to evaluate and assess the model-updating practices of AI and ML clinical models that are used in direct patient-provider clinical decision-making.

**Methods:**

We used the PRISMA (Preferred Reporting Items for Systematic Reviews and Meta-Analyses) checklist and the PRISMA-P protocol guidance in addition to a modified CHARMS (Checklist for Critical Appraisal and Data Extraction for Systematic Reviews of Prediction Modelling Studies) checklist to conduct this scoping review. A comprehensive medical literature search of databases, including Embase, MEDLINE, PsycINFO, Cochrane, Scopus, and Web of Science, was conducted to identify AI and ML algorithms that would impact clinical decision-making at the level of direct patient care. Our primary end point is the rate at which model updating is recommended by published algorithms; we will also conduct an assessment of study quality and risk of bias in all publications reviewed. In addition, we will evaluate the rate at which published algorithms include ethnic and gender demographic distribution information in their training data as a secondary end point.

**Results:**

Our initial literature search yielded approximately 13,693 articles, with approximately 7810 articles to consider for full reviews among our team of 7 reviewers. We plan to complete the review process and disseminate the results by spring of 2023.

**Conclusions:**

Although AI and ML applications in health care have the potential to improve patient care by reducing errors between measurement and model output, currently there exists more hype than hope because of the lack of proper external validation of these models. We expect to find that the AI and ML model-updating methods are proxies for model applicability and generalizability on implementation. Our findings will add to the field by determining the degree to which published models meet the criteria for clinical validity, real-life implementation, and best practices to optimize model development, and in so doing, reduce the overpromise and underachievement of the contemporary model development process.

**International Registered Report Identifier (IRRID):**

PRR1-10.2196/37685

## Introduction

### Background

The ubiquitous application of artificial intelligence (AI) and machine learning (ML) algorithms in health care delivery has increased [1]. Investments in clinical AI and ML algorithms are based on their perceived potential to improve health care quality [1]. These algorithms can automate insights directly from data without using standard computer programming [2]. In addition, they can analyze large data sets with high dimensionality to yield insights and predictions on complex associations without prior assumptions from traditional statistical methods, differentiating AI and ML models from other statistical techniques (interference statistics, classical prediction models, and hypothesis testing) [3,4]. Generally, 2 methods of learning from data exist in AI and ML: supervised and unsupervised learning. Supervised learning involves making predictions based on a set of prespecified input, references, and output variables, whereas unsupervised learning is used to draw inferences from data sets consisting of input data without labeled responses [5].

There has been a paradigm shift in health care stakeholders’ goals of quality improvement in recent years, with an emphasis on achieving better outcomes at lower costs, while improving the efficiency of care delivery and prioritizing personalized care [6]. This change, resulting in the use of AI and ML algorithms, has also been driven by regulators and payers demanding high-value care rather than volume-based care, as well as the changing role of patients as consumers [7]. In addition, the unprecedented abundance of data with the advent of electronic health records (EHRs) and other direct consumer wearables allows the application of data-heavy clinical models [8].

Despite these perceived potentials, AI and ML algorithm performance degrades over time, particularly because of model calibration (calibration drift), which refers to the accuracy of risk estimates in terms of agreement between the predicted risks of events and their actual observed frequencies [9]. Calibration drift arises as a consequence of deploying a model in a dynamic environment, with the resulting difference between the population or setting in which the model was trained and that in which it was implemented [10].

Degradation of a model over time can also occur within the same health care system where it was derived [11]. Among other factors, there is a tendency toward a systematic data shift when a model is successfully deployed [12,13]. The downstream characteristics of data change owing to differences in the distribution of outcomes in a prognostic model as users respond to the model prediction. The more effective a predictive model is to improve outcomes, the faster the model will likely degrade [14].

Because of the sensitive nature of patient-level algorithm predictions, consistency and accuracy are critical. Therefore, an appropriate model-updating process is essential across the lifetime of the model [15]. Model updating aims to improve the performance of an existing model by adjusting (recalibrating) its parameters and predictors, either within the same clinical environment in which the model was developed or within an external environment [15,16]. The best practice is to update a clinical model rather than abandon the model, build another, or repeat the selection of predictors, which leads to a loss of the previous scientific information captured [11,17].

The existence of multiple models for the same clinical scenario without model-updating methods declared ab initio leaves clinicians uncertain of which model is appropriate to use, potentially resulting in adverse consequences for patient care [17]. For example, there are more than 80 models for the prognosis of stroke [18], more than 20 models predicting intensive care unit stay after cardiac surgery [19], more than 100 published algorithms for prognosis after neurotrauma [20], and over 50 models to predict outcomes after breast cancer [21].

### Peculiarities and Challenges of Model Updating in the Health Care Environment

The health care model updating process faces unique challenges owing to the health care sector’s dynamic clinical, environmental, and regulatory ecosystem [22]. Therefore, it is imperative to consider all these issues from the early stages of clinical model development to ensure consistency and accuracy over time [1,23].

Subtle population demographic changes, in addition to changes in health care access and the heterogeneity of health insurance coverage (health disparity), can also deteriorate a model’s future output [1,24]. Changes in best practice clinical guidelines, in addition to variations in practice preferences across different health care providers, can also be a source of data shift, resulting in suboptimal model output [1,15,25-29].

Health centers often update or change information systems and digital health tools such as imaging software and EHRs. Models that are not updated based on the data output of new information systems will be suboptimal [1]. In addition, there is a constant change in clinical nomenclature and disease coding, which can also affect the output [23].

The health care regulatory landscape is constantly evolving as well [30,31]. The enactment of the Affordable Care Act, which was associated with many sweeping reforms to health care delivery and redefining value in health care delivery, rendered previous standards of care invaluable [31]. As such, a model built based on those standards will likely be suboptimal.

### Issues Regarding Health Care Model Input Data

Learning artifacts or bias specific to the sites where training data sets were produced or because of the nature of the data set itself can ultimately result in a data set shift and model degradation over time [1]. Most AI tools are developed based on the nuances of specific local health care workflows and the data they generate. For example, consider an algorithm developed to predict sepsis based on a patient’s lactate level. The algorithm will learn to correlate the physician’s lactate orders with a high possibility of sepsis. However, model quality would be reduced if there were a policy change requiring more frequent ordering of lactate tests.

Model validation in these circumstances shows reduced performance, as the learned pattern does not generalize across sites and circumstances [24,32]. In addition, there is systemic bias in the geographic distribution of patient cohorts, as algorithms trained on US data were disproportionately trained on patients from just 3 states (New York, California, and Massachusetts) [33].

Label and causality leakage phenomena occur when the model’s prediction target is directly or indirectly present in the training data set, rendering the model prediction irrelevant [34]. An example is a model developed to predict hospital mortality in patients admitted to the intensive care unit. An AI model trained naively on all data will learn to correlate extubating and turning off the ventilator with the death of a patient and ultimately produce a near-perfect predictive performance yet with absolutely no clinical utility [34]. Causality leakage in the clinical model can occur in a situation whereby a clinician orders a test based on a high index of suspicion of a clinical outcome that the algorithm is meant to predict; the algorithm then uses the test to generate an alert that results in an action [35].

### Overview of Model Updating Methods in Health Care

There are several methods that address the data shift required to update models [1,16,17,23,36]. Although extensive details of these methods are beyond the scope of our analysis, we have highlighted the most important methods here [16,17,23,36]. The least complex method involves adjusting the model intercept to a different prevalence or incidence rate according to the new population (assuming risk factors still confer the same level of risk). Another option is adjusting the population prevalence rate and adding a single adjustment to all risk factors in the model. One or more risk factor relationships may also need to be adjusted, given the changes in relationships over time. A more drastic method involves adjusting both the prevalence and the coefficients and adding new risk factors into the model.

The last option involves refitting the entire model based on a new data sample, either alone or in combination with the addition of new potential risk factors (essentially remodeling the problem from scratch on a new sample). The best options typically depend on the time from initial model development to the time sample sizes are updated [23]. With larger samples or longer time periods since the initial fitting, ideal updates usually involve the prevalence updating option or refitting the model based on the updated sample. With small samples, it is generally advised that no updates are made. With shorter periods of time since the last update, it is generally recommended that the prevalence be updated.

### Time and Frequency of Model Updating

A few approaches exist to guide the timing and frequency of model updating, each with its own advantages and limitations. Real-time calibration drift detection and updating is usually the most computationally intensive approach; however, real-time detection provides users with the peace of mind that their models are accurate at the time of use without requiring manual steps [23]. A similar approach is incremental updating, in which models are updated based on new instances as they become available [16,23]. This approach is computationally extensive and requires the same infrastructure to automatically provide near-real-time updates automatically. Fixed and batch updating at specified intervals is another option, with models evaluated and updated at specific intervals; however, if the frequency of the update is not ideal, model drift issues may exist before the update [16,23].

### Study Objectives

Our main study objective is to evaluate model updating in AI and ML clinical models and assess model updating practices used in direct patient-provider clinical decision-making. Previously published reviews have established that most clinical AI and ML models do not conduct external validation of their models [5]. In addition, phases of model development pertaining to applicability and reproducibility (model updating, impact assessment, and implementation) have received less attention in the scientific literature [37]. Clinical model-updating processes seek to prevent model deterioration with adverse consequences, such as inaccuracy or lack of practicality in clinical settings; model updating can also impact generalizability and reproducibility [37]. The model-updating processes of clinical algorithms must be determined proactively from the time of initial model development [32,37] to ensure patient safety and quality of care. Understanding the degree to which model updating is prioritized will help inform the validation of future models and guide the modification of best practices in model development.

Our intentions are as follows:

1. To determine if the process of clinical model updating is mentioned or prioritized in the reviewed published clinical AI studies used to support direct patient-provider clinical decision-making.
2. To determine if AI and ML studies in the literature include demographic data and if there are significant geographic distributions of models whose investigators recommended model-updating procedures in their publications.
3. To test correlations between the quality of published AI clinical models and prioritization of the model-updating process.

## Methods

### Eligibility Criteria: Inclusion and Exclusion

This study protocol is for a scoping review. Our original protocol was developed based on the PRISMA (Preferred Reporting Items for Systematic Reviews and Meta-Analyses) Protocols [38].

#### Inclusion Criteria and Rationale

Only AI and ML studies that involve clinically predictive or prognostic modeling used to support specific clinical decisions by medical providers for or against intervention for direct patient care will be included. In addition, only human studies with algorithms applied to organic and emotional and behavioral health domains used directly in addressing clinical problems (by supporting patient-provider decision-making) will be included. Examples of algorithms we will include are those predicting outcomes that affect clinical decisions and treatment (mortality and morbidity and predicting length of hospital stay) and those predicting complications and health improvement.

Algorithms comparing diagnostic modalities and tools with the possibility of affecting clinical decisions also fall under our inclusion criteria. We will include both observational and experimental studies regardless of study methods, as there is a dearth of randomized controlled trials (RCTs) in AI and ML studies owing to the novelty of AI applications in health care [39].

Supervised ML methods, including both classification and regression methods, will also be included. Most supervised predictive model outputs directly impact decisions at the point of use, unlike unsupervised or clustering and semisupervised methods, mostly to generate insights for a predictive problem [40]. Studies will be included without geographic or regional preferences. All studies that were published from March 2018 until March 2021 will be reviewed (Textbox 1).

**Inclusion criteria.**

- Inclusion criteria and definition
  Only artificial intelligence and machine learning studies that involve clinically predictive or prognostic modeling: Diagnostic prediction models calculate an individual’s risk of having an illness, whereas prognostic prediction models calculate the risk of certain health conditions that could occur in the future.
  Study outcome and outcome measures format: Only human studies with algorithms and outcomes in organic and emotional or behavioral health domains. All study outcome measures format will be included as follows: continuous, binary, ordinal, multinominal, and time-to-event.
  Supervised machine learning technique: Only articles with supervised learning methods will be included, with methods such as regression, ensemble, and decision trees.
  Study design and data source: Any experimental or observational study that meets our inclusion criteria will be included. These include randomized controlled trials, prospective and retrospective cohorts, case-control studies, and case-cohort studies. All data sources are permitted, including data registries and electronic health record data.
  Predictors: Articles that use at least two predictor variables in their model development will be included.

#### Exclusion Criteria

Health care AI models that do not use clinical domains as a primary end point (studies of health care operations, finance, billing, and inventory management) will be excluded.

AI and ML studies whose predictions may not directly support provider-patient clinical decision-making, including the following, will also be excluded.

- Studies of population-based estimates only (incidences, prevalence, and others)
- Patient or provider satisfaction with care
- Studies designed to improve diagnostic tools, such as imaging and genomics
- Imaging biosignal studies that do not directly impact clinical decision-making
- Studies that evaluate health care system quality indicators

Unsupervised and semisupervised learning and clustering studies will also be excluded, as most data mining and unsupervised learning models are used to generate insights into a problem or identify predictive modeling problems [40]. Reviews, articles, commentaries, letters to the editor, conference abstracts, and commentary articles without algorithms will also be excluded.

We will exclude models embedded in proprietary software where the specific ML methods used are not specified. Failure to meet any of the above eligibility criteria will result in exclusion from the review (Textbox 2).

**Exclusion criteria.**

- Exclusion criteria and definition
  Health care artificial intelligence studies that do not involve clinical domains as the primary end point: Studies of health care operations, finance and billing, and inventory managements. These do not fall primarily under patient treatment and care.
  Purpose and potential of study not directly supporting provider-patient clinical decision-making: Studies that may not impact direct provider decision-making at the point of care, such as population-based estimates only (incidences and prevalence), those designed to improve diagnostic tools, and those that evaluate health care system quality indicators.
  Unsupervised learning and clustering studies: Those that mostly are used to generate insight into a problem or identify predictive modeling problems.
  Nonexperimental articles and proprietary models: Reviews, articles, commentaries, letters to the editor, abstracts, commentaries without algorithms, and models embedded in a proprietary software whereby the specific machine learning methods used are not specified.
  Genomics and advanced genetic algorithms: These articles are usually based on very high dimensional data and unsupervised methods, which are beyond the scope of our analysis.
  Pathological specimen and image signal studies: Pathological specimen studies mostly seek to improve accuracy at the level of the clinical pathologist. Image signals studies are mostly used to improve the accuracy of an imaging instrument rather than provide a basis for preferred clinical outcomes.

### Information Sources

A comprehensive literature search will be conducted using the following databases: Ovid Embase, Ovid MEDLINE, Ovid PsycINFO, Web of Science Core Collection, Scopus, and the Cochrane Library. Searches will be limited to articles published from January 1, 2018, to December 31, 2021.

### Search Strategy

The search strategy for each database was developed by a medical librarian (SW) in concert with the rest of the team. Each search strategy used a combination of keywords and subject headings related to ML, predictive algorithms, medical diseases and disorders, and study design (Multimedia Appendix 1).

### Statistical Analysis

#### Data Management

All search results will be imported into Covidence software for deduplication and screening [41]. Covidence facilitates a blind review process, and results from multiple databases can be imported, deduplicated, and screened for eligibility. Following the title and abstract screening phase, the full text of all included abstracts will be gathered and imported. Covidence will create a PRISMA flowchart and facilitate the data extraction and quality appraisal phases [42].

#### Selection Process

**Figure 1:**
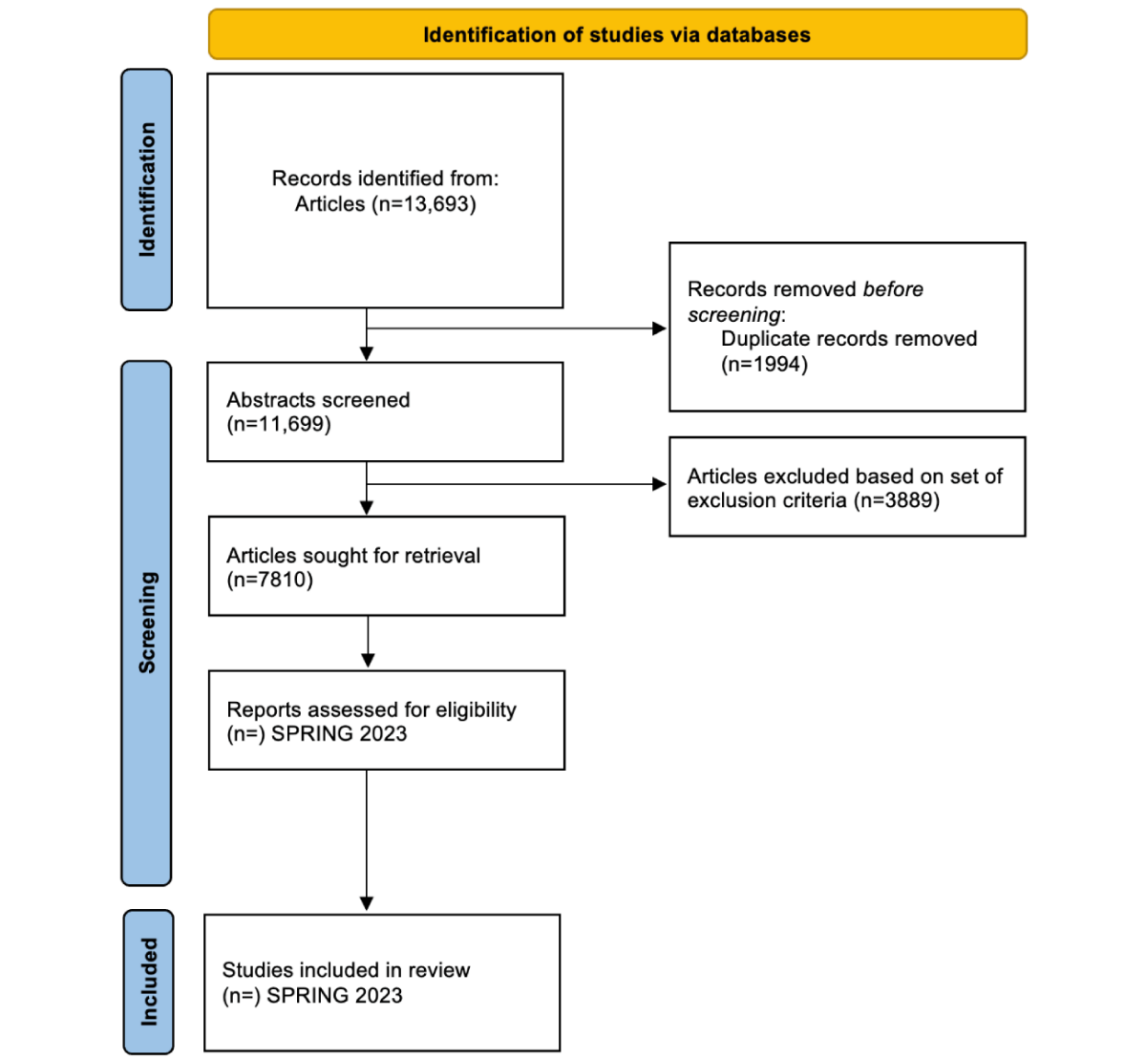
PRISMA flow chart.

Two reviewers will use the Covidence software to independently screen the title and abstract of each article and the full text of all included abstracts. A third-party independent reviewer will resolve disparities. The screening process will be documented and presented using the PRISMA flow diagram like the flowchart in progress in Figure 1.

Before title and abstract screening, the review team will meet to screen a random sample of 50 records to validate the inclusion and exclusion criteria.

#### Data Collection Process

Variables collected from each eligible study will be guided by the CHARMS (Checklist for Critical Appraisal and Data Extraction for Systematic Reviews of Prediction Modelling Studies) [43] and PRISMA checklists [42].

#### Data Items

Data items will reflect the objective of our study, which is the need to prioritize clinical model updating as an integral part of validating published medical algorithms. Therefore, our data items will pertain to generalizability and applicability.

We will collect general study information, such as the title; first author; year of publication; clinical setting, such as academic, nonacademic, vendor, and governmental agency; disease system of the study; aim of the algorithm (predictive vs prognostic); type of algorithm (traditional ML vs neural network or deep learning); and geographic region. We will also collect data on study type and model type (research vs production model) as well as model-updating methods.

Our preliminary search on geographic clusters of AI adoption and model implementation revealed that AI and ML adoption is mostly clustered in the United States, Canada, the United Kingdom, Australia, the European Union, China, Taiwan, and Israel [44,45]. As these regions account for most of the clinical models created, we will use another category of “Other” to capture models developed in other geographic regions. We will also collect information about the data sources such as EHRs, open registry or open sources, and closed registry or proprietary databases.

In the *Methods* section, we will abstract the data relating to the assessment of study quality (Table 1). Data on handling missing data will also be abstracted, such as sample size, well-defined primary outcomes, and predictors. We will also abstract data on the study limitations considered in the Discussion section of the article in review.

**Table 1:** Checklist for Critical Appraisal and Data Extraction for Systematic Reviews of Prediction Modelling Studies.

Domains and key items			General		Applicability		Risk of bias
**Source of data**							
	Source of data (cohort, case-control, randomized trial participants, or registry data)			✓		✓	
**Participants**							
	Participant eligibility and recruitment methods (consecutive participants, location, number of centers, setting, and inclusion and exclusion criteria)	✓		✓			
	Participant descriptions	✓		✓			
	Details of treatment received, if relevant			✓		✓	
	Study dates	✓		✓			
**Outcome to be predicted**							
	Definition and methods for outcome measurements			✓		✓	
	Determine if the same outcome definition and method for measurement was used in all patients					✓	
	Type of outcome (single or combined end points)	✓		✓			
	Determine if the outcome was assessed without knowledge of candidate predictors (blinded)					✓	
	Determine if candidate predictors were part of the outcome (in panel or consensus diagnosis)					✓	
	Time of outcome occurrence or summary of duration of follow-up			✓			
**Candidate Predictors (or Index Test)**							
	Number and type of predictors (demographics, patient history, physical examination, additional testing, and disease characteristics)	✓					
	Definition and method for measuring candidate predictors			✓		✓	
	Timing of predictor measurement (patient presentation, diagnosis, and treatment initiation)			✓			
	Determine if predictors were assessed blinded for outcome and for each other (if relevant)					✓	
	Handling predictors in the modeling (continuous, linear, and nonlinear transformation or categorized)					✓	
**Sample size**							
	Number of participants and number of outcomes or events	✓					
	Number of outcomes or events in relation to the number of candidate predictors (events per variable)					✓	
**Missing data**							
	Number of participants with any missing values (including predictors and outcomes)	✓				✓	
	Number of participants with missing data for each predictor					✓	
	Handling of missing data (complete case analysis, imputation, or other methods)					✓	
**Model development**							
	Modeling methods (logistics, survival, neural networks, or machine learning techniques)	✓					
	Modeling assumptions satisfied					✓	
	Method for selecting predictors for inclusion in multivariable modeling (all candidate predictors and preselection based on unadjusted association with the outcome)					✓	
	Methods for selecting predictors during multivariable modeling (full model approach backward or forward selection) and criteria used (*P* value and Akaike Information Criterion)					✓	
	Shrinkage of predictor weights or regression coefficients (no shrinkage, uniform shrinkage, and penalized estimation)			✓		✓	
**Model performance**							
	Calibration (calibration plots, calibration slope, and Hosmer-Lemeshow test) and discrimination (*C*-statistic, *D*-statistic, and log-rank) measures with CIs			✓			
	Classification measures (sensitivity, specificity, predictive values, and net reclassification improvement) and whether a priori cut points were used					✓	
**Model evaluation**							
	Method used for testing model performance: development data set only (random split of data, resampling methods, bootstrap or cross-validation, or none) or separate external validation (temporal, geographic, different settings, and different investigators)					✓	
	In case of poor validation, whether the model was adjusted or updated (intercept recalibrated, predictor effects adjusted, or new predictors added)			✓		✓	
**Results**							
	Final and other multivariable models (basic, extended, and simplified) presented, including predictor weights or regression coefficients, intercept, baseline survival, and model performance measures (with SEs or CIs)	✓		✓			
	Any alternative presentation of the final prediction models (sum score, nomogram, score chart, and predictions for a specific risk subgroup with performance)	✓		✓			
	Comparison of the distribution of predictors (including missing data) for development and validation data sets					✓	
**Interpretation and discussion**							
	Interpretation of presented models (confirmatory, model useful for practice vs exploratory, and more research needed)	✓		✓			
	Comparison with other studies, discussion of generalizability, strengths, and limitations	✓		✓			

#### Evaluation Outcomes

##### Primary Outcome

The primary outcome of this scoping review is the percentage of published algorithms that prioritize model-updating methods (model updating is considered prioritized if it is part of the algorithm protocol). We also identify articles that mention model updating but do not apply it to algorithm protocols.

##### Secondary Outcomes

We will also identify any correlations between prioritizing model updating and geographic region, and quality of studies, as well as temporal correlation and correlation by setting of model development. In addition, we will assess how frequently EHRs are used for model development, given the high incidence of inaccuracies in EHR data [46-48].

As a secondary end point, we will capture the incidence of models reporting the demographic breakdown of their data (ethnic background and gender); this is of particular importance owing to potential societal harm and resulting AI and ML algorithm setbacks because of the use of nonrepresentative data [49].

#### Quality of Studies and Risk of Bias Assessment

Owing to the overemphasis on model technical validity at the expense of downstream clinical validity in published algorithms, a clinical model that has acceptable, technically valid prediction results during model development with favorable statistical indexes, such as area under the curve, does not automatically translate to model effectiveness when deployed in real-life clinical scenarios [1,50-52]. Rather than the in-depth technical validity of model results, our evaluation of individual model quality will assess a model’s ability to attain high real-life clinical validity for generalizability. Our goal is to focus on established factors and best practices that indicate a study’s applicability and low risk of bias to ensure generalizability beyond the model’s technical output as follows [52-54]:

- Applicability: the extent to which the study fits within the inclusion and exclusion criteria of the review
- Risk of bias: the extent to which any flaws in the study lead to overly optimistic estimates of predictive performance measures (CHARMS article)
- Generalizability: the degree to which the study results are relevant to the larger population
- Reproducibility: the ability to duplicate the study using the same methods used in the original study

#### Checklist/Evaluation Tools for Study Quality Assessment

Best practices recommend adequate reporting of model development to ensure reproducibility and applicability of models in real clinical settings [2]. To evaluate the quality of reporting of the reviewed published models, we used an adaptation of a verified tool available for model quality assessment [43]. The CHARMS is an 11-item checklist, with each item created to assess the model study on the domains of risk of bias and applicability (Table 1). The checklist is a comprehensive guide created from a combination of 8 other published guides that include both criteria to ascertain applicability and reproducibility with implications for patient safety, as well as technical validity of a model’s results, some of which are beyond the scope of our review.

We created our quality assessment tool by extracting the criteria that are more specific to applicability and reproducibility analysis and that have a potential impact on patient safety and quality of care at the level of clinical model deployment, which resulted in our 6-item checklist for study quality assessment (Table 2). A total of 5 items out of our 6-item checklist were adapted from the CHARMS checklist; our last criteria, the model development checklist standard, was obtained from literature review best practices for model development.

**Table 2:** Quality of studies and risk of bias assessment.

Assessment criteria				Maximum score (stars)
**Study design and missing data**				
	**Study design**			
		RCTs^a^	(**)	
		Other sources and designs; cohorts, registries, convenient sampling	(*)	
	Handling of missing data		(*)	
**Outcome**				
	Primary outcome is well defined		(*)	
**Model testing and evaluation methods**				
	Separate external validation data; geographical, temporal, and population		(**)	
	Same development data used for validation; random split and reassembly (bootstrap and cross-validation)		(*)	
**Model updating method**				
	Yes		(**)	
**Model interpretation and applicability concerns**				
	Strengths and weaknesses of model (reproducibility, applicability, or risk of bias)		(*)	
**Model reporting and development standard**				
	Best practice standard for model development and reporting defined; examples of standards: CONSORT-AI^b^, SPIRIT-AI^c^, DECIDE-AI^d^, NEUR-UPS ML^e^, TRIPOD-ML^f^, PROBAST-ML^g^, and STROBE^h^		(*)	

^a^RCT: randomized controlled trial.

^b^CONSORT-AI: Consolidated Standards of Reporting Trials-Artificial Intelligence.

^c^SPIRIT-AI: Standard Protocol Items: Recommendation for Interventional Trials- Artificial Intelligence.

^d^DECIDE-AI: Developmental and Exploratory Clinical Investigation of a Decision-Support System Driven by Artificial Intelligence.

^e^NEUR-UPS ML: Neural Informational Processing System in Machine learning.

^f^TRIPOD-ML: Transparent Reporting of a Multivariable Prediction Model of Individual Prognosis Or Diagnosis-Machine Learning.

^g^PROBAST-ML: Prediction model Risk Of Bias Assessment Tool- Machine Learning.

^h^STROBE: The Strengthening the Reporting of Observational Studies in Epidemiology.

#### Rationale for Checklist Items

##### Checklist Items Adapted From the CHARMS Checklist: Study Design and Data Source for Model Development

The data used to develop the algorithm may be sourced from retrospective and prospective cohorts including RCTs and cross-sectional studies. In addition, there is a proliferation of sourcing model data from registries, databases, and EHRs. Although RCTs are considered the gold standard, they also have shortcomings similar to all other methods. Although RCTs are designed to reduce biased outcomes, their findings can lead to impaired generalizability of outcomes in real-life clinical scenarios owing to the rigid eligibility criteria of study participants [43]. Data sources for model development are critical for the predictive accuracy, applicability, and reproducibility of any algorithm [11,12,43,50,51].

- Outcomes: the lack of well-defined study outcomes increases risk of bias and adversely affects model reproducibility in real-life clinical scenarios [43]. For example, 40% of cancer prognostic model studies were found to have poorly defined outcomes [55]. For our quality assessment, a well-defined outcome is considered to occur when the definition and measurement of the outcome events or target disease clearly correspond to the outcome definition of the study objective [43].
- Model testing and evaluation methods: model validation is the process of quantifying model performance in other individuals beyond the training and testing data set used to develop the model [56]. Whenever the predictive performance of a model is estimated using the same data set that was used to develop the model, it is referred to as “apparent performance” [43]. Regardless of which modeling technique is used, apparent performance tends to be biased, as it can overestimate performance relative to the performance of other individuals. It is very important that all models be evaluated in an independent data set (external validation) before deployment [55]. Externally validated models (either temporal or geographic validation) provided the best insights into the usefulness of the model for other individuals, centers or settings, and regions. Several reviews have shown that external validation studies are generally uncommon [5,20,57,58], as most studies are only internally validated by a random split sample of the data into development and validation samples [5]. Because of the higher impact of external validation on model applicability in real-life clinical scenarios, we prioritize these models in our checklist by allocating 2 stars to any study with externally validated models (Table 1)
- Model updating method recommendation: in the event that an existing model shows poor performance when evaluated in other settings (geographic or temporal), it is best practice to adjust, update, or recalibrate the original model to increase performance [43], as there are well-established methods to achieve successful model updating. It is also best practice that the potential techniques for updating a model on external deployment can be identified before deployment [1,32]. The primary outcome of our review is the proactive determination of possible model-updating methods. As such, we will prioritize any study that proactively suggests a model-updating method as part of its study method by scoring it as 2 stars.
- Model interpretation and generalizability concerns: best practice guidelines for reporting medical studies recommend discussing strengths, weaknesses, and future challenges with regard to the generalizability of the studies [59-61]. For models, these studies should therefore provide insight into the model’s applicability, usefulness, and intended users [43]. This discussion also serves as a basis for comparison with other studies. Therefore, our quality checklist will include a score (1 star) for a study that mentions the strengths and weaknesses of their model in the Discussion section.

##### Other CHARMS Checklist Items

The remaining 6 items in CHARMS were excluded from our assessment tool because they were already considered during the initial screening stage of our review process (participant characteristics and predictors). We also excluded items that focused on technical assessment, as that is beyond the scope of our study objective of real-life clinical applicability (technical process of model development, model performance, results, and sample size). Although the checklist still needs to be validated, our adapted checklist captures the essence of our review.

#### Checklist Items Based on a Literature Review of Best Practices of Clinical Model Studies: Model Development Reporting Standards

The best practice standards for reporting primary prognostic and predictive model studies exist in the literature [62] and include SPIRIT-AI (Standard Protocol Items: Recommendation for Interventional Trials- Artificial Intelligence), CONSORT-AI (Consolidated Standards of Reporting Trials-Artificial Intelligence), TRIPOD (Transparent Reporting of a Multivariable Prediction Model of Individual Prognosis Or Diagnosis), REMARK (Reporting Recommendations for Tumour Marker Prognostic Studies), and GRIPS (Genetic Risk Prediction Studies) [63-67]. Adhering to these guidelines may ensure study reproducibility and could improve future real-life applications [62,63,68]. Despite the availability of these guidelines, there is poor overall quality of reporting in many published AI models [53,62,68,69]. Therefore, we have included declaring a reporting standard as part of our checklist (reporting standard scores will receive 1 star).

For each checklist item fulfilled by the study reviewed, studies will be scored with 1 or 2 stars as described above, with a maximum score of 10 stars each.

#### Data Synthesis

After extracting data from the manuscripts, we will conduct a narrative synthesis. Data will be summarized using descriptive statistics, figures, and tables for visualization. Categorical data will be presented through numbers and percentages. The distribution of continuous data such as sample size and the number of predictors will be assessed and described using means and SDs for normally distributed data using median and 25th and 75th percentiles for nonnormally distributed data. The results will be characterized by study design, outcomes, service delivery type, ML techniques, and model-updating properties.

#### Ethics Approval

On August 13, 2021, our systematic review protocol was registered with the International PROSPERO (Prospective Register of Systematic Reviews) CRD42021245470 [70]. Our protocol was developed based on the PRISMA-P (Preferred Reporting Items for Systematic Reviews and Meta-Analysis Protocols) 2015 statement [38]. Our study does not require an ethics committee review because our research does not directly involve human subject data and it will be conducted on publicly available data from published articles.

## Results

So far, we have conducted a literature search of the specified databases. We are now in the title and abstract screening phase. Our initial literature search yielded 13,693 articles; after removing duplicates, we obtained approximately 11,699 articles. We identified approximately 7810 articles for full article review among the 8 reviewers (Figure 1). We hope to complete the review process and disseminate the review results by spring of 2023.

## Discussion

### Principal Findings

AI and ML applications in health care are significantly increasing at an estimated 40% compounded annual growth rate [71]. Most models are proliferating because of their perceived potential for increased quality of health care at the point of care by providing real-time clinical decision support, early warning sign systems, clinical documentation, improved administrative workflow, medical device automation, and better imaging analysis [2,71,72]. Their implementation has the potential to move the needle from a reactive to a proactive approach, focusing on health management rather than disease treatment [71]. Despite this potential, there is a lack of adequate external validation and real-life assessment of the applicability of these models [5,36], which can adversely affect the generalizability of clinical models at the point of implementation [5,73]. There is also concern regarding algorithmic bias and worsening health inequity.

Owing to the complex nature of health care environments, clinical algorithms tend to deteriorate over time. Considering the constantly evolving nature of medical practice in response to new technology, epidemiology, and social phenomena, it appears we will always be chasing a moving target with regard to outcome prediction using an algorithm [72]. Therefore, the relevance of clinical data as predictor factors decays with a half-life of only 4 months [74]. This decay phenomenon reinforces the need for model-updating methods that can adapt to evolving data from the inception of model development [74]. Although there are proposed methods for model updating in the literature [23], a lack of inclusion of these methods in published algorithms can impair a model’s applicability and reproducibility. This review aims to highlight and raise awareness of these issues to encourage model developers to improve their protocols.

### Limitations

Interpretation of our review should bear some limitations in mind. First, AI and ML implementations in health care are relatively novel and lack standardization across different regions and clinical specialty domains. Although we established our literature search strategy (Multimedia Appendix 1), this lack of standards can impact the scope and sensitivity of our search and render the reproducibility of our review challenging. While the terms “AI” and “ML” are included in our search, terms used to describe models and modeling are not standardized, and therefore, it is possible that our strategy will not capture possible emerging or lesser-known terms. In addition, our search included only English language publications, and, as such, we cannot generalize our findings to publications in other languages. In addition, we did not include book chapters, theses, short papers, editorials, non–peer-reviewed reports, or conference abstracts.

Another factor to consider in the interpretation of our results is that the studies we reviewed were published during the global COVID-19 pandemic. The impact of the pandemic on the nature and type of AI and ML studies published during this time is unknown.

### Conclusions

In this scoping systematic review, we will review published AI and ML algorithms across all clinical fields and geographic regions to determine how frequently model-updating methods are suggested in published studies. We believe that the AI and ML model-updating methods offered in published models are a proxy for a model’s generalizability and implementation reproducibility. We aim to determine the geographic distribution of published models that prioritize model-updating methods and if any correlations exist between the quality of the model reported and the suggested model-updating method. Owing to the faulty evaluation of real-life generalizability and reproducibility of AI systems, recent studies have shown that health care AI and ML performance may be overly optimistic [72]. Although AI and ML applications in health care have potential, some have argued that AI and ML is presently riding atop the peak of “inflated expectations” [72]. We aim to ascertain the degree to which published model results include ethnic and gender demographic data in the light of well-established algorithmic bias in health care.

Our findings will add to the literature on model clinical validation and real-life implementation and help improve best practices for model development by prioritizing updating. We will conduct the scoping review with the hope of moving the needle of contemporary model development away from the peak of “inflated expectations” [72] to the nadir of enlightened reality.

## Appendix

Multimedia Appendix 1 Complete search strategy.

## Abbreviations

AI: artificial intelligence
CHARMS: Checklist for Critical Appraisal and Data Extraction for Systematic Reviews of Prediction Modelling Studies
CONSORT-AI: Consolidated Standards of Reporting Trials-Artificial Intelligence
EHR: electronic health record
GRIPS: Genetic Risk Prediction Studies
ML: machine learning
PRISMA: Preferred Reporting Items for Systematic Reviews and Meta-Analyses
PRISMA-P: Preferred Reporting Items for Systematic Reviews and Meta-Analysis Protocols
PROSPERO: Prospective Register of Systematic Reviews
RCT: randomized controlled trial
REMARK: Reporting Recommendations for Tumour Marker Prognostic Studies
SPIRIT-AI: Standard Protocol Items: Recommendation for Interventional Trials- Artificial Intelligence
TRIPOD: Transparent Reporting of a Multivariable Prediction Model of Individual Prognosis Or Diagnosis
