# Supplementary material for: The Need to Prioritize Model-Updating Processes in Clinical Artificial Intelligence (AI) Models: Protocol for a Scoping Review
Source: JMIR Res Protoc. 2023 Feb 16;12:e37685. doi: 10.2196/37685 (PMC9982723; doi:10.2196/37685)
Supplement: Multimedia Appendix 1 [file resprot_v12i1e37685_app1.pdf]

Multimedia Appendix 1: Complete Search Strategies

Ovid Embase

1. exp machine learning/ or exp artificial intelligence/

2. (machine learning or artificial intelligence or deep learning).mp.

3. 1 or 2

4. ((predictive or prediction or prognostic or forecasting) adj2 (algorithm* or model)).mp.

5. Random forest algorithm*.mp.

6. Boosted regression algorithm*.mp.

7. Support vector machine.mp.

8. Neural network algorithm*.mp.

9. logistic regression algorithm*.mp.

10. Pattern recognition.mp.

11. Time Series Prediction.mp.

12. (Ensemble adj3 algorithm).mp.

13. 4 or 5 or 6 or 7 or 8 or 9 or 10 or 11 or 12

14. 3 and 13

15. exp mental disease/ or exp physical disease/

16. 14 and 15

17. patient*.mp.

18. 16 and 17

19. exp controlled clinical trial/

20. exp clinical study/

21. (cross-sectional or observational or prospective or retrospective or randomized or randomized or quality improvement).mp.

22. 19 or 20 or 21

23. 18 and 22

24. limit 23 to yr="2009 -Current"

Ovid Medline

1. exp machine learning/ or exp artificial intelligence/

2. (machine learning or artificial intelligence or deep learning).mp.

3. 1 or 2

4. ((predictive or prediction or prognostic or forecasting) adj2 (algorithm* or model)).mp.

5. Random forest algorithm*.mp.

6. Boosted regression algorithm*.mp.

7. Support vector machine.mp.

8. Neural network algorithm*.mp.

9. logistic regression algorithm*.mp.

10. Pattern recognition.mp.

11. Time Series Prediction.mp.

12. (Ensemble adj3 algorithm).mp.

13. 4 or 5 or 6 or 7 or 8 or 9 or 10 or 11 or 12

14. 3 and 13

15. exp mental disorders/ or exp "diseases (non mesh)"/

16. 14 and 15

17. patient*.mp.

18. 16 and 17

19. exp Epidemiologic Study Characteristics/

20. exp Study Characteristics/

21. (cross-sectional or observational or prospective or retrospective or randomized or randomized or quality improvement).mp.

22. 19 or 20 or 21

23. 18 and 22

24. limit 23 to yr="2009 -Current"

Ovid PsycINFO

1. exp machine learning/ or exp artificial intelligence/

2. (machine learning or artificial intelligence or deep learning).mp.

3. 1 or 2

4. ((predictive or prediction or prognostic or forecasting) adj2 (algorithm* or model)).mp.

5. Random forest algorithm*.mp.

6. Boosted regression algorithm*.mp.

7. Support vector machine.mp.

8. Neural network algorithm*.mp.

9. logistic regression algorithm*.mp.

10. Pattern recognition.mp.

11. Time Series Prediction.mp.

12. (Ensemble adj3 algorithm).mp.

13. 4 or 5 or 6 or 7 or 8 or 9 or 10 or 11 or 12

14. 3 and 13

15. (cross-sectional or observational or prospective or retrospective or randomized or randomized or quality improvement).mp.

16. exp experimentation/

17. 15 or 16

18. 14 and 17

19. limit 18 to yr="2009 -Current"

Cochrane Library

(machine learning or artificial intelligence or deep learning)

AND

(((predictive or prediction or prognostic or forecasting) NEAR/2 (algorithm* or model))

OR

("Random forest algorithm*" or "Boosted regression algorithm*" or "Support vector machine" or "Neural network algorithm*" or "logistic regression algorithm*" or "Pattern recognition" or "Time Series Prediction")

OR

(Ensemble adj3 algorithm))

Limit to: 2009 -Current

Web of Science Core Collection

TS=(machine learning or artificial intelligence or deep learning)

AND

TS=(((predictive or prediction or prognostic or forecasting) NEAR/2 (algorithm* or model))

OR

("Random forest algorithm*" or "Boosted regression algorithm*" or "Support vector machine" or "Neural network algorithm*" or "logistic regression algorithm*" or "Pattern recognition" or "Time Series Prediction")

OR

(Ensemble NEAR/3 algorithm))

AND

TS=(Patient OR patients)

AND

TS=(cross-sectional or observational or prospective or retrospective or randomized or randomized or quality improvement or pilot)

Limit to: 2009-present

Scopus

(machine learning or artificial intelligence or deep learning)

AND

(((predictive or prediction or prognostic or forecasting) W/2 (algorithm* or model)) OR

("Random forest algorithm*" or "Boosted regression algorithm*" or "Support vector machine" or "Neural network algorithm*" or "logistic regression algorithm*" or "Pattern recognition" or "Time Series Prediction") OR (Ensemble W/3 algorithm))

AND

(Patient OR patients)

AND

(cross-sectional or observational or prospective or retrospective or randomized or randomized or quality improvement or pilot)

Limit to: 2009-present
